# Long-term impacts of untreated dairy manure on the microbiome and Shiga toxin-producing *Escherichia coli* persistence in agricultural soil

**DOI:** 10.1128/aem.00447-25

**Published:** 2025-08-07

**Authors:** Taylor K. S. Richter, Michael Kauffman, Mark K. Mammel, David W. Lacher, Gireesh Rajashekara, Susan R. Leonard

**Affiliations:** 1 Office of Applied Microbiology and Technology, Human Foods Program, U.S. Food and Drug Administration, Laurel, Maryland, USA; 2 Department of Animal Sciences, College of Food, Agricultural, and Environmental Sciences, The Ohio State University 155699 , Wooster, Ohio, USA; University of Georgia Center for Food Safety, Griffin, Georgia, USA

**Keywords:** *Escherichia coli*, foodborne pathogens, environmental microbiology, metagenomics

## Abstract

Biological soil amendments of animal origin (BSAAO) improve the soil health of agricultural fields for plant growth. However, as natural reservoirs for bacterial foodborne pathogens, BSAAO application can introduce and support microbes of public health concern, such as pathogenic Shiga toxin-producing *Escherichia coli* (STEC), in agricultural soils. Using shotgun metagenomic sequencing, this project investigated the microbiome of soil with and without BSAAO, focusing on STEC and the *E. coli* population over time alongside changes in the soil microbiome and soil abiotic properties. Two farms in Ohio, one using an untreated dairy manure amendment and one that does not use a BSAAO, were sampled for over a year for metagenomic analysis of the soil microbiome. All manure samples were positive for *stx* genes, indicating the presence of STEC. Impacts of the manure on the soil lasted four weeks by several measures including higher *E. coli* diversity and more frequent STEC detection. Outside of these four weeks post-amendment, Shiga toxin genes (*stx*) were identified periodically in both fields throughout the year. STEC detection significantly correlated with higher *in silico E. coli* O-serogroup diversity, as well as lower soil cation exchange capacity and concentrations of calcium, magnesium, and organic nitrogen. Differential abundance analysis of the soil metagenomes identified several taxa influenced by amendment but did not identify taxa correlated with detection of *stx* genes. This work provides insights into the timing of and ecological factors associated with STEC persistence in the agricultural environment.

**IMPORTANCE:**

Shiga toxin-producing *E. coli* (STEC), including *E. coli* O157:H7, is a major etiological agent of foodborne human disease outbreaks associated with fresh produce and can be transferred to produce via contaminated agricultural soil. Given the devastating impacts of foodborne STEC outbreaks on public health and growers, it is necessary to understand the longevity of the impacts of manure application on the pathogen risk in the soil as well as better understand the ecological and environmental conditions that contribute to STEC survival in the agricultural soil environment. This work expands upon the knowledge of conditions that support STEC persistence in the produce-growing environment and its longevity following amendment in commercial fields with naturally occurring STEC contamination.

## INTRODUCTION

Soil amendments are used to improve agricultural soil conditions for plant growth. The use of biological soil amendments of animal origin (BSAAOs) has the added benefit of disposing of accumulated animal waste (1, 2). BSAAOs vary in their degree of processing, ranging from untreated manure to fully composted material that has reached time and temperature requirements necessary for pathogen reduction (3). While BSAAOs of all treatment types improve conditions for crop growth by increasing essential soil nutrients, water-holding capacity, and soil organic matter, they vary in production time, cost, and microbiological risk (1, 2). BSAAOs can increase soil microbial diversity through both the input of nutrients and the introduction of manure microbes into the soil (4–6). However, animal manure is a natural source of foodborne pathogens, and its use as a soil amendment can introduce or support human pathogens in the crop-growing environment (7, 8).

Shiga toxin-producing *Escherichia coli* (STEC), such as *E. coli* O157:H7, is one of the leading causes of foodborne illness globally (9). STEC infection causes gastrointestinal illness but can progress to hemolytic uremic syndrome (HUS), a serious condition that may require hospitalization and can be fatal (10, 11). Shiga toxin has two major variants, Stx1 and Stx2, each with several known subtypes that are associated with varying disease severity (11–13). In addition to the Shiga toxins, STEC can possess several other virulence factors, including intimin (encoded by *eae*), enterohemolysin (*ehxA*), autoagglutinating adhesin (*saa*), and subtilase cytotoxin (*subAB*) (10, 11). The virulence genes *saa* and *sub*AB are typically carried in *eae*-negative STEC (14). *E. coli* O157:H7 is the most common and well-studied STEC serotype accounting for most STEC infections in the United States and is the source of several major food-related outbreaks around the world (9, 10, 15–17). Yet the number of O-serogroups associated with STEC disease outbreaks is increasing, including the so-called “big six” non-O157 serogroups (O26, O45, O103, O111, O121, and O145), which have been responsible for most of the outbreaks in the United States and Europe (16, 18). STEC infection typically occurs through consumption of contaminated food, with several large outbreaks associated with leafy greens (17, 19–21). With the goal of preventing STEC illness, research has included the ecology of STEC persistence in the agricultural environment and mechanisms of produce contamination (22–26).

Fecal contamination of the produce-growing and processing environment (such as the soil, water, or workers’ hands) is a known source of STEC on fresh produce (25). The animal gastrointestinal tract is a natural reservoir for *E. coli*, with STEC found in animals including wildlife and livestock, such as cattle, where it colonizes without causing disease to the animal (26–31). Cattle and cattle manure have been implicated as a source of STEC contamination in fields (24, 32, 33). Still, cattle manure continues to be a valuable soil amendment for farmers in the United States, particularly on smaller-scale and organic farms (34, 35). To mitigate the risks of transferring human pathogens to crops through BSAAO usage, current guidelines from the U.S. Food and Drug Administration recommend a waiting period of 90 or 120 days, depending on whether the edible portion of the plant is in contact with the ground—between BSAAO application and product harvest (3). The waiting period is to allow time for potential foodborne pathogen populations to decline prior to harvest. However, there is a need for more science-based data on the longevity of the microbiological impacts of BSAAO-amended soils, including naturally occurring pathogen survival (35).

In this study, we used metagenomics to survey the long-term effects of BSAAO on STEC prevalence and diversity, as well as on the microbiome in agricultural soils. Samples were collected from two small specialty farms in Ohio, one which applied raw, untreated dairy manure as a soil amendment and the other which applied no BSAAO. Any potential STEC contaminants found in the raw dairy manure would have been from naturally occurring origination. Soil was sampled for over a year following the application of the amendment, and associations between STEC detection and farm practices, weather conditions, edaphic features, and the soil microbiome were investigated. This research improves our understanding of the impacts of BSAAOs on the soil microbial environment and the efforts to support food safety practices in produce-growing operations.

## RESULTS

### Field and soil environment

Two agricultural fields were chosen for sample collection due to their proximity and different amendment practices used. Field B received an amendment of bedded pack manure, consisting of a combination of raw dairy heifer manure, straw, and corn stalks. Field N did not receive a BSAAO. Both fields were part of limited-mechanization, small specialty crop farms growing a variety of produce that were weeded either by hand or with horse-drawn weeder. To cover the entire time between annual soil amendment application, samples were collected over the course of a year (Table 1). Sampling began in 2020 before being interrupted by the COVID-19 pandemic and restarted in 2021. In both years, manure amendment was added in February. Planting in 2021 began in April, while all 2020 sampling took place with no crops in the field. In the summer of 2021, field B was planted with zucchini, cabbage, and peppers, while field N was planted with onions, peppers, green beans, melons, and sweet corn. Planting and harvesting of crops occurred on a rolling basis throughout the spring, summer, and fall. Given the proximity, the fields experienced similar weather conditions (Table S1). While precipitation was highest in May and July, snow accumulation in the winter meant that the amendment was applied to the field on top of a layer of snow.

**TABLE 1:** Sample collection timeline showing days post-amendment (dpa)^*a*^

Month	2020		2021		2022	
	dpa	Sample collected	dpa	Sample collected	dpa	Sample collected
January	−32	B, N	−8	B, N	337	B, N
February	−1	B, N	−1	B, N	367	B
	1	B, M	1	B, N, M		
	3	B	20	B, N		
	5	B				
	7	B				
	9	B				
March	11	B	28	B		
	14	B	42	B, N		
	18	B	52	N		
	21	B	56	B		
April			70	B, N		
			72	B		
			74	B		
			77	B		
			81	B		
May			90	B, N		
June			120	B, N		
			142	B, N		
July			177	B, N		
August			198	B		
September			225	B, N		
October			254	B		
November			282	B, N		
December			309	B		

^*a*^ B, soil from the amended field; N, soil from the unamended field; M, manure amendment.

**Fig 1:**
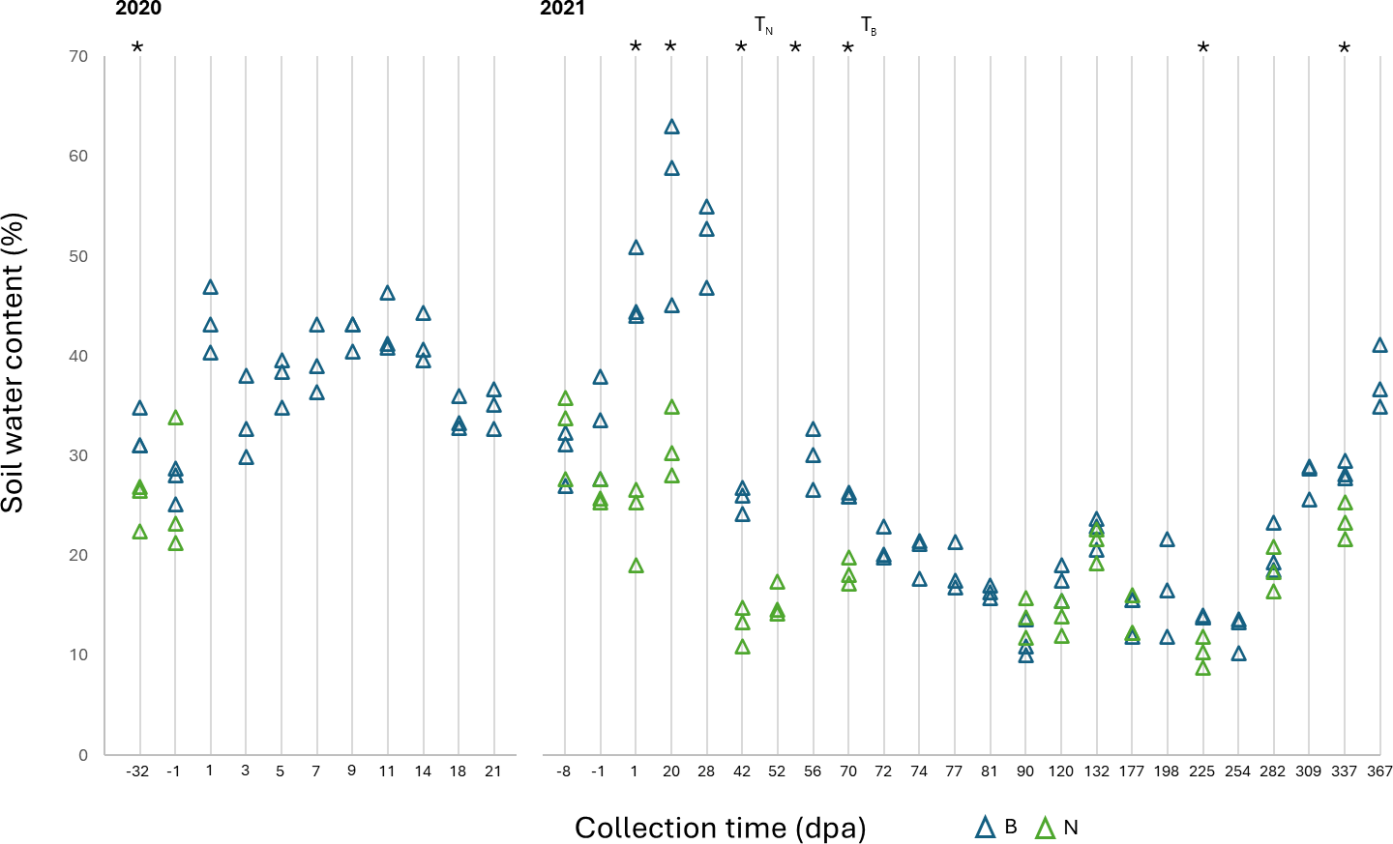
Soil moisture is shown as percent water content in samples collected from field B (amended, blue markers) and field N (unamended, green markers). Asterisks (*) identify time points (shown as days post-amendment, dpa) at which the soil samples from fields B and N significantly differ (Student’s *t*-test, *P* < 0.05). The timing of soil tilling on field N (*T*_*N*_) and field B (*T*_*B*_) is denoted.

**Fig 2:**
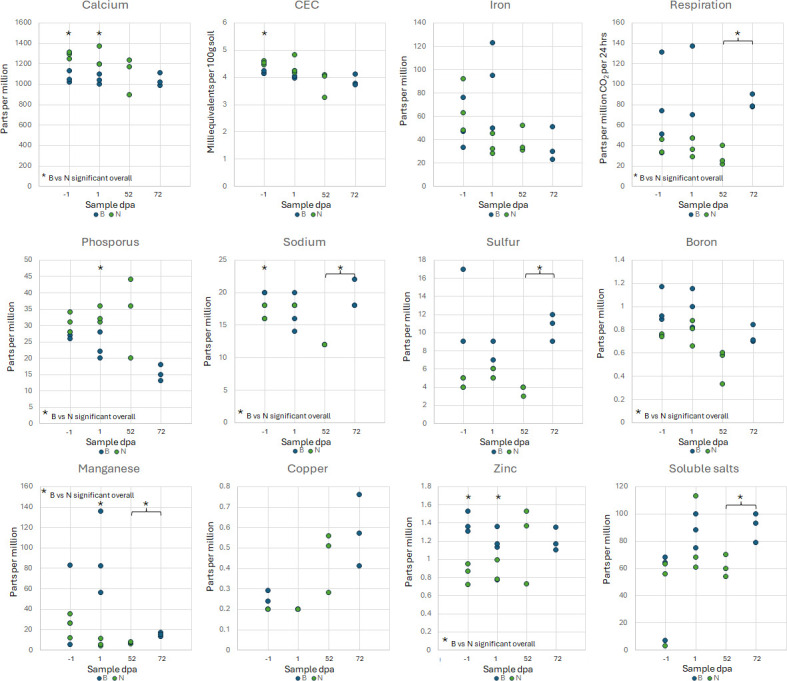
Summary of the nutrient profiles of soil samples collected in 2021 from field B (amended, blue markers) and field N (unamended, green markers). Asterisks (*) identify time points (shown as days post-amendment, dpa) at which the measurement means significantly differ between fields (Student’s *t*-test, *P* < 0.05). Details and measurements from 2020 are shown in Table S1.

Soil physical and chemical properties were measured periodically throughout the course of the study. The soil was classified as loam and loam/silt loam on fields N and B, respectively. The moisture content was significantly higher in the amended compared to unamended soil starting the day after amendment and remained significantly higher than the unamended field for at least 70 days, persisting after tilling (Student’s *t*-test, *P* < 0.05; Fig. 1). The pH in the two fields varied throughout the year, but rarely diverged significantly from one another, nor were there apparent changes in pH associated with amendment or tilling (Table 2). Organic carbon concentration was significantly higher in the soil in field B compared to field N overall (Student’s *t*-test, *P* = 0.004), although they rarely differed significantly at individual time points (Table 2). Similarly, organic nitrogen concentration was significantly higher in field B overall (Student’s *t*-test, *P* = 0.011), with one exception in which field N had a higher concentration immediately after tilling at 52 dpa, which occurred 20 days prior to tilling in field B. Pre-amendment, post-amendment, and post-tilling samples from both fields were used for more in-depth soil property measurements (Fig. 2; Table S2). Overall, field B had significantly higher soil respiration (as measured by CO_2_ released in a 24-hour period), as well as higher concentrations of organic matter and magnesium, and soluble salts were significantly higher in field B soil, but only in the post-tilling samples (Student’s *t*-test, *P* < 0.05). Additionally, soils from field B had significantly higher concentrations of a greater number of soil nutrients—namely, organic carbon and nitrogen, sodium, sulfur, boron, manganese, and zinc—throughout the year, whereas field N had significantly higher concentrations of only phosphorus and calcium (Fig. 2; Table S2).

**TABLE 2:** Summary of the soil pH, organic nitrogen concentration (ppm), and organic carbon concentration (ppm), listing averages of replicates days post-amendment (dpa) in field B (amended) and field N (unamended)^*a*^

Year	dpa	pH		Organic nitrogen		Organic carbon	
		B	N	B	N	B	N
2020	−1	7.36^a,f^	7.02^a,g^				
2021	−1	6.66	7.48^g^	16.00^p^	12.20	190.50	168.10^v^
	1	7.42	7.26	17.00^k^	11.20^q^	199.73	137.13^v,w^
	42	7.00	6.97	86.60^i,k,l^	39.30^i,q,r^	138.90^t^	110.77^t,w^
	52		6.98		10.27^r,s^		128.20
	70	7.50^b^	6.62^b^	30.10^l^	45.47^s^	175.90^u^	112.97^u^
	72	7.19		16.00^m^		139.30	
	142	6.55^d^	6.55	54.63^m,n^	44.50	152.97	133.80^x^
	177	6.00^d^		126.53^n^		163.80	
	198	6.00		129.60		164.60	
	282	6.25	6.53^h^	76.20	35.70	244.77	191.80^x,y^
	337	6.47^c,e^	7.10^c,h^	49.07^j,o,p^	16.57^j^	164.60	157.37^y^
	367	7.13^e,f^		11.70^o^		144.33	

^*a*^ Letters designate statistical significance between corresponding groups of samples (Student’s *t*-test, *P* < 0.05).

### STEC detection

**Fig 3:**
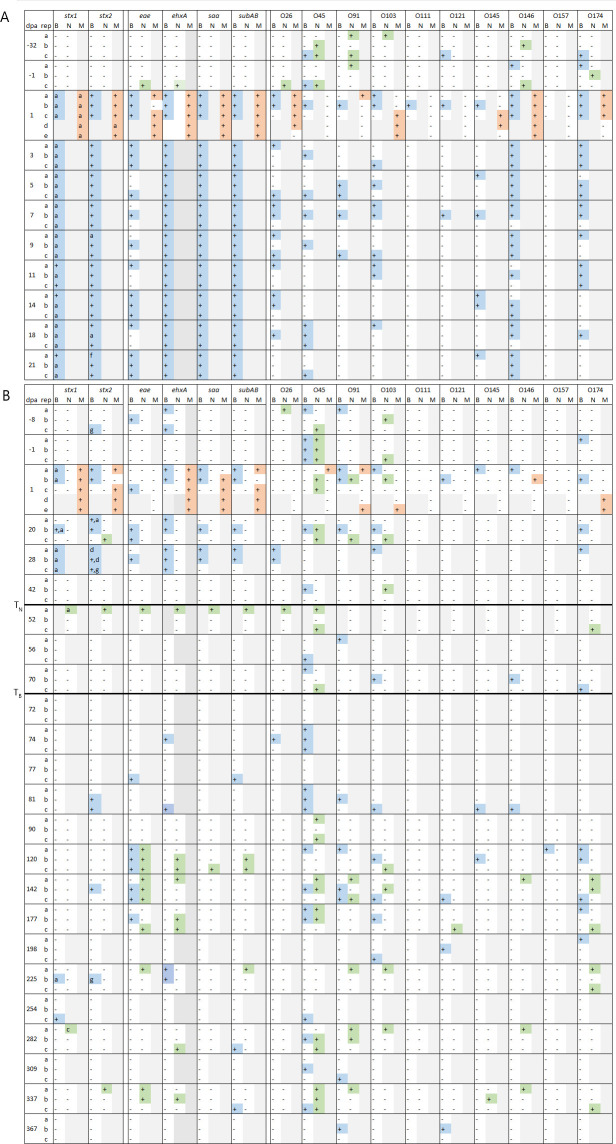
Presence (+) or absence (−) of STEC-related virulence and O-serogroup genes in enriched metagenomes from soil and manure samples in (**A**) 2020 and (**B**) 2021, shown by days post-amendment (dpa) and replicate (rep). *Stx* subtypes are labeled by letter when identifiable; undistinguished subtypes are marked with a +. Gray boxes indicate where no sample was collected. Colored boxes show positive detections by sample type: blue (field B, amended), green (field N, unamended), and orange (manure). Read counts are shown in Tables S3 and S4. The timing of soil tilling on field N (*T*_*N*_) and field B (*T*_*B*_) is also denoted with a bold line.

STEC presence in a sample was determined by the detection of any one of the genes responsible for Shiga-toxin production, namely, *stx1* and *stx2* and their subtypes, in the enrichment metagenomes. *E. coli* toxins and O-serogroup genes could not be detected in the culture-independent (CI) metagenomes; therefore, soil samples underwent an overnight enrichment for *E. coli* prior to metagenomic sequencing. In the enrichment metagenomes, several O-serogroups and *stx* subtypes, including *stx1a*, *stx1c*, *stx2a*, *stx2d*, *stx2f*, and *stx2g*, were detected (*stx2b* and *stx2c* were not detected). A total of 54 samples were positive for STEC, including 100% (10/10) of the manure samples, 39.2% (40/102) field B soil samples, and 8.3% (4/48) field N soil samples (Fig. 3). STEC genes were detected more often in soil samples in the first four weeks following amendment application, 94.4% (34/36) samples overall and in 100% (27/27) and 77.8% (7/9) of the samples from 2020 and 2021, respectively. Outside these four weeks post-amendment, STEC-positive soil samples were identified at the same frequency, 9.5%, in both fields B (6/63) and N (4/42) in 2021. Both *stx1* and *stx2* genes were often detected in the same sample, although in 5.6% (3/54) samples, only *stx1* was detected, and in 13.0% (7/54), only *stx2* was detected (Fig. 3B). The majority of the *stx1* genes were *stx1a* (36 samples). Only one sample had *stx1c*, and in 11 samples, the subtype could not be identified due to low coverage. While most samples had *stx2* genes whose subtypes could not be distinguished (46 samples), subtypes *stx2a* (4 samples), *stx2d* (2 samples), *stx2f* (one sample), and *stx2g* (3 samples) were also identified. Since sample B25c was STEC-positive and had an anomalously high number of reads in the metagenome, we sought to determine if STEC detection was a function of sequencing depth. To do this, we compared read numbers to *stx* gene detection but found no correlation. Although *stx* genes were used as a proxy for STEC presence, other STEC genes encoding virulence factors, namely, *eae*, *ehxA*, *saa*, and *subAB*, were also detected in this study, most frequently in *stx-*positive samples. Of the 54 *stx*-positive samples, 29 also carried *eae*, 49 carried *ehxA*, 43 carried *saa*, and 43 carried *subAB* (Fig. 3; Table S3). Conversely, in the 106 *stx-*negative samples, 19 were positive for *eae*, five were positive for *ehxA*, one was positive for *saa,* and six were positive for *subAB* (Fig. 3; Table S3). As with the *stx* genes, the STEC virulence factors were found consistently during the four weeks post-amendment, as well as sporadically throughout the year in 2021. Only *eae* was particularly enriched in the months around harvest (June–August) compared to the months before (Fisher’s exact test, *P* < 0.0001) and after (Fisher’s exact test, *P* = 0.0013) (Table S5). There were also significantly more *eae-*positive samples during harvest than during the four weeks post-amendment (Fisher’s exact test, *P* = 0.012).

### STEC presence correlated with environmental factors

While the strongest predictor of STEC presence in the soil was time post-manure amendment application, STEC genes were detected in soils that were not amended by BSAAO. Several edaphic measurements were taken periodically throughout the study to determine possible correlation with STEC presence (Table 3). Water content was significantly correlated with *stx* detection (rank biserial correlation, two-tailed, *r*_rb_ = 0.76, *P* < 0.0001), with higher water content (average 36.8%, range 13.7–63.0%) in *stx-*positive soil compared to *stx-*negative soil (average 22.44%, range 8.75–45.13%). However, soil water content was no longer significantly correlated with STEC presence when samples from the four weeks post-amendment were excluded. STEC gene detection was further correlated with lower soil concentrations of calcium (average of 978 ppm and 1,162 in *stx-*positive and *stx*-negative soils, respectively; rank biserial correlation, two-tailed, r_rb_ = −0.82, *P* < 0.0001), magnesium (average 140 ppm and 161.33 ppm in *stx-*positive and *stx*-negative soils, respectively; rank biserial correlation, two-tailed, *r*_rb_ = −0.67, *P* = 0.002), and organic nitrogen (average 23.53 ppm and 45.98 ppm in *stx-*positive and stx-negative soils, respectively; rank biserial correlation, two-tailed, *r*_rb_ = −0.32, *P* = 0.01), as well as with a decreased cation exchange capacity (average 3.2 meq/100 g soil in *stx-*positive soils and 4.21 meq/100 g soil in *stx-*negative soils; rank biserial correlation, two-tailed, *r*_rb_ = −1, *P* < 0.0001). Weather factors were not significantly correlated with the detection of STEC genes.

**TABLE 3:** Soil characteristic correlations with STEC detection showing the average for each measure in STEC-positive vs STEC-negative soils^*a*^

Soil characteristic (units)	Average in STEC-positive soil	Average in STEC-negative soil	*r*_rb_	*P*
pH	6.79	6.64	−0.09	0.49
**Water content (%)**	**36.80**	**22.44**	**0.76**	**<0.0001**
Respiration (ppm CO_2_/24 hrs)	82.23	54.61	0.38	0.12
Organic C (ppm)	156.75	156.08	0.10	0.47
**Organic N (ppm)**	**23.53**	**45.98**	**−0.32**	**0.01**
**C:N ratio**	**10.13**	**6.84**	**0.43**	**0.0008**
Nitrate (ppm)	2.47	5.00	−0.02	0.93
Ammonium (ppm)	0.52	0.26	−0.29	0.24
*P* (ppm)	22.67	28.07	−0.42	0.08
K (ppm)	326.00	226.27	0.31	0.21
**Ca (ppm)**	**978.00**	**1162.27**	**−0.82**	**<0.0001**
**Mg (ppm)**	**140.00**	**161.33**	**−0.67**	**0.002**
Na (ppm)	15.33	17.33	−0.42	0.08
S (ppm)	6.00	7.13	−0.13	0.60
B (ppm)	0.83	0.79	0.24	0.33
Fe (ppm)	83.00	46.87	0.47	0.05
Mn (ppm)	74.67	21.53	0.42	0.08
Cu (ppm)	0.23	0.33	−0.24	0.33
Zn (ppm)	1.07	1.13	−0.13	0.90
**Cation exchange capacity (meq/100** **g)**	**3.20**	**4.21**	**−1.00**	**<0.0001**
Loss on ignition (%)	3.20	3.63	0.04	0.86
Soluble salts (ppm)	80.67	65.35	0.22	0.37

^*a*^ Edaphic features significantly correlated with STEC detection (rank biserial correlation, *P* < 0.05) are shown in bold.

### *E. coli* community diversity, virulence, and correlations with the environment

Changes to the overall *E. coli* community, along with correlations to environmental factors and STEC detection, were also studied. *E. coli* diversity was measured based on the number of O-serogroups detected in the enrichment metagenomes, and Wilcoxon rank sum test was used to compare the median number of serogroups between groups of samples. The number of O-serogroups found in soil ranged from two to 164 and four to 121 in fields B and N individual replicates, respectively, and the median number of serogroups did not differ significantly between fields prior to amendment (Fig. S1; Tables S4 and S5). Following amendment, field B soil samples had a significantly higher median *E. coli* diversity compared to pre-amendment soil (3.7-fold higher in 2020, *P* = 0.0004; 1.8-fold higher in 2021, *P* = 0.034) and relative to N field soil (2.9-fold higher in 2021, *P* = 0.012). This increase remained for four weeks following amendment in 2021 and then returned to its pre-amendment levels (Fig. S1). Sampling in 2020 was suspended before returning to pre-amendment levels. The amendment samples had higher median number of serogroups than in pre-amendment soil in both years (median 24.5 serogroups in 2020 soil, 124 in 2020 amendment, 23 in 2021 soil, and 43 in 2021 amendment), though this difference was only significant in 2020 (*P* = 0.023). The median number of O-serogroups in the amendment was significantly higher in 2020 than in 2021 (median of 124 and 43 O-serogroups, respectively; *P* = 0.012), as was the resulting increase in soil *E. coli* diversity following amendment (median of 91 in 2020 and 42 in 2021) (Fig. S1; Table S5). Significant differences in read numbers between the 2020 and 2021 samples were not observed. Thus, the differences in the number of detected O-serogroups cannot be explained by differences in sequencing depth (Table S6). Spearman correlations with soil and environmental factors revealed that high O-serogroup richness in samples taken outside the four weeks post-amendment was correlated with high organic carbon concentration (ρ = 0.48, *P* = 0.0012), low concentration of soluble salts (ρ = −0.78, *P* = 0.00048), low precipitation (ρ = −0.24, *P* = 0.039), and low wind speed (ρ = −0.21, *P* = 0.031) (Table S7). Significantly higher serogroup diversity was also found in STEC-positive soils, which had a median of 77 O-serogroups compared to STEC-negative soils, with a median of 19 O-serogroups (Wilcoxon rank sum, *P* < 0.00001). Similarly, in unamended soil samples from the N field and from the B field outside of the four weeks post-amendment, STEC-positive soils had twice the median O-serogroup diversity as STEC-negative soils (median 36 and 18 serogroups, respectively; Wilcoxon rank sum, *P* = 0.00138).

### Non-STEC *E*. *coli* virulence genes in the soil environment

Virulence genes corresponding to other *E. coli* pathotypes were also identified in the enrichment metagenomes from both fields, including both the amended and unamended soil from field B (Fig. S2). The gene encoding the ETEC heat-labile toxin was the most prevalent virulence factor found in the soils, identified in 78.8% of the field B soil samples in 2020 and 33.3% in 2021. In field N soil, the gene was detected in 33.3% and 26.2% of the samples in 2020 and 2021, respectively. The second most prevalent gene was *cdtB*, observed in 78.8 and 21.7% of field B soil in 2020 and 2021, respectively, and 50 and 21.4% in field N soil in 2020 and 2021, respectively. While these non-STEC virulence genes were overall significantly enriched in amended samples, only two genes*—cdtB,* found in multiple pathotypes, and LT-II, from ETEC—were significantly enriched in amended soils compared to field B soils prior to and after the four weeks post-amendment and to field N soils (Fig. S2; Table S5). As with the STEC virulence factors, these other *E. coli* virulence genes were overall significantly enriched in the summer months compared to other seasons. However, only *cdtB* demonstrated significant enrichment in the summer when examined individually.

### Core *E. coli* community in soil and manure

*E. coli* was detected in all enrichment metagenomes, with at least four O-serogroups identified, suggesting a “resident” *E. coli* population in the soil. A total of 240 O-serogroups were identified, but the *E. coli* O-serogroup population was variable over time and within sample groups, making it difficult to identify “core” members of the *E. coli* community (Tables S8 and S9). Several O-serogroups were more likely to occur in soil from the four weeks post-amendment relative to unamended soil, and in manure compared to soil, as determined by Fisher’s exact test (*P* < 0.05; Table S9). Serogroup O101 was significantly more likely to occur in soils than in manure and was one of the most commonly and consistently identified O-serogroups in both fields in 2021. STEC-positive soils had significantly higher detection frequencies of 35 O-serogroups in field B, 27 of which were also found in higher frequencies in amended soils (compared to unamended) and manure (compared to soil) (Table S9). In field N soils, 16 O-serogroups were found in higher frequencies in *stx*-positive soils, six of which (O15, O91, O102, O130, O159, and O179) were shared by *stx*-positive samples in both fields (Table S9). Eleven serogroups (O22, O32, O109, O112ab, O123, O150, O156, O159, OXY34, OXY55, and OXY65) were more common in both the amended soils and manure. However, these O-serogroups are not exclusive to the amended soils, or even field B, suggesting that dairy manure amendment is not the only source of these *E. coli*.

### Changes in the culture-independent soil microbiome

**Fig 4:**
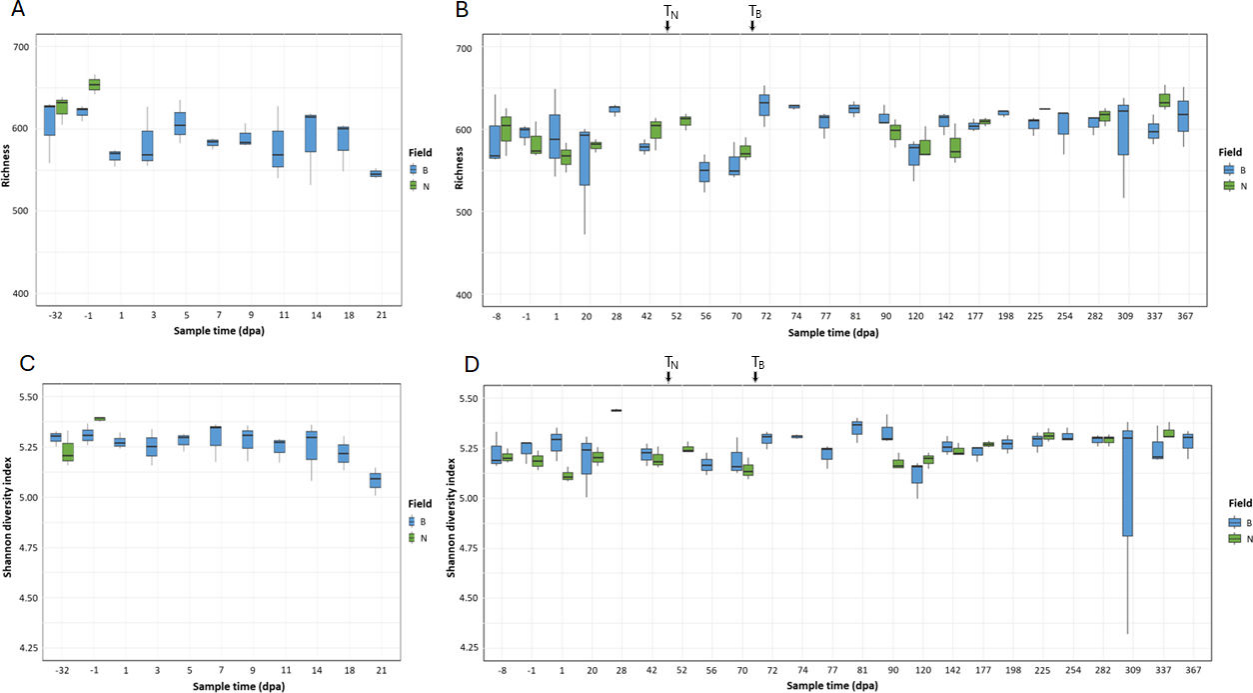
Alpha diversity of the culture-independent soil metagenomes from field B (amended, blue bars) and field N (unamended, green bars) by days post-amendment (dpa). Richness, measured as the number of taxa, is shown in the (**A**) 2020 soil samples and (**B**) 2021 soil samples. Shannon diversity is shown for (**C**) the 2020 soil samples and (**D**) the 2021 soil samples. The timing of soil tilling on field N (*T*_*N*_) and field B (*T*_*B*_) is denoted.

The culture-independent (CI) metagenomes were investigated to determine the impacts of amendment and STEC presence on the soil microbiome. In 2020, field B richness significantly decreased following amendment (Fig. 4A; Wilcoxon rank sum, *P* = 0.0235), though a similar decline following amendment was not observed in 2021 (Fig. 4B). Shannon diversity increased during the four weeks post-amendment (1–28 dpa) in field B relative to field N, averaging 5.297 in field B and 5.158 in field N (Wilcoxon rank sum, *P* = 0.039) (Fig. 4C and D). STEC-positive soils displayed a small, though significant, increase in Shannon diversity compared to STEC-negative soils, with median Shannon indices of 5.29 and 5.23, respectively (Wilcoxon rank sum, *P* = 0.0178)

**Fig 5:**
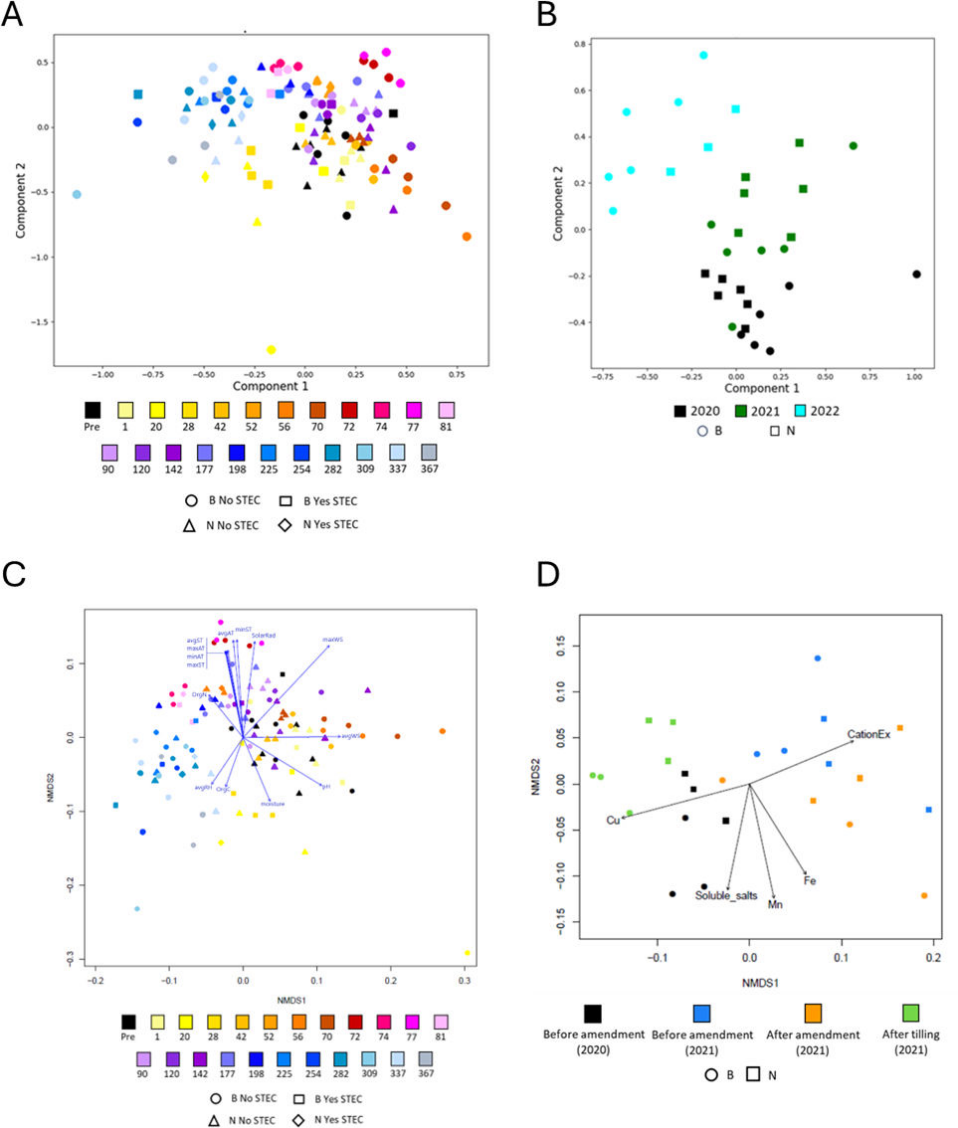
Beta diversity analyses of soil microbial communities and correlations with environmental factors. (**A**) NMDS plot of Bray–Curtis dissimilarity for culture-independent (CI) soil communities in fields B and N in 2021, with pre-amendment samples in black. (**B**) NMDS plot showing CI communities in January and February of 2020, 2021, and 2022. (**C**) NMDS plot of 2021 CI communities colored by days post-amendment (dpa), with vectors indicating significant environmental correlations (PERMANOVA, *P* < 0.05), including soil moisture, pH, organic carbon (OrgC), organic nitrogen (OrgN), air temperature (AT), soil temperature (ST), relative humidity (RH), solar radiation (SR), and wind speed (WS). (**D**) NMDS plot of beta diversity at key time points (pre-amendment, one dpa, and post-tilling) showing significant correlations with soil chemistry: cation exchange capacity (CationEx), iron (Fe), manganese (Mn), copper (Cu), and soluble salts.

The major soil taxa remained relatively consistent over time and between fields (Fig. S3) without significant changes due to amendment. The soil was dominated by diverse members of the Proteobacteria, followed by Actinobacteria. The manure samples were dominated by the genus *Pseudomonas*. While the relative abundance (RA) of *Pseudomonas* increased for several soil samples following amendment, the changes were not significant. Beta diversity analyses were used to examine changes in the soil microbial communities following the addition of the manure amendment and over time. Neither the manure-amended soils nor STEC-positive soils had distinct microbial communities compared to other samples (Fig. 5A). The soil microbial communities changed with time, with samples demonstrating distinct microbial communities by dpa throughout the year (Fig. 5A; PERMANOVA, *P* = 0.0001) and in winter samples over three years (Fig. 5B; PERMANOVA, *P* = 0.0001).

The envfit function of the vegan software package was used to determine the correlation between soil microbial composition and environmental variables (Fig. 5C and D). Several weather and soil properties—namely, soil moisture, soil temperature, air temperature, pH, organic carbon and organic nitrogen concentration, solar radiation, wind speed, and average relative humidity—were significantly (*P* < 0.05) correlated with soil microbial composition (Fig. 5C). Only precipitation, minimum and maximum relative humidity, and average wind direction were not identified as significantly correlated. A similar analysis was performed using soil chemistry from select samples. In this analysis, 19 soil health and nutrient variables were tested, but only five (cation exchange capacity and the concentrations of iron, manganese, copper, and soluble salts) were significantly correlated (*P* < 0.05) with microbiome composition (Fig. 5D). As with the environmental factors, the importance of the significant soil chemical factors varied with the timing of sample collection.

### Correlations between amendment, soil microbiota, and STEC presence

We investigated the differentially abundant (DA) taxa in amended versus unamended soils to identify individual soil taxa correlated with manure amendment. Only pre-amendment samples and samples from the four weeks post-amendment were compared to maintain consistency between years and reduce the impacts of warming temperatures during spring and summer on taxonomic changes. *Pseudomonas caeni* was the most significantly differentially abundant taxa in amended soils in both 2020 and 2021 (Fig. S4A and S4B). It was the most abundant taxa in the manure, ranging from 16 to 64% RA, and was not detected in the soil prior to amendment, suggesting a transfer from the amendment to the soil community. Only two other species—*Clavibacter michiganensis* and *Microbacterium foliorum*, both in the Microbacteriaceae family—were significantly enriched in amended soils in both 2020 and 2021. The Microbacteriaceae taxa were in higher RA in the pre-amendment soil (ranging from 2 to 4% RA in 2021) than in the manure amendment (from 0.5 to 0.8% RA in 2021), suggesting that these taxa were not introduced to the soil by the manure but benefitted from the improved soil conditions following amendment. The significantly enriched taxa in amended soil represent a small portion of the soil microbial community, totaling approximately 5% combined RA.

Correlations between differentially abundant taxa and STEC presence were less clear than those with amendment. Soil samples in which STEC was detected outside of the four weeks post-amendment were of particular interest. Field B had six such STEC-positive samples, but differential abundance analysis of these and STEC-negative soils identified no significant taxa correlated with STEC presence. Field N had four STEC-positive soil samples throughout the year, and several taxa were identified as significantly enriched in STEC-positive soils (Fig. S4C), including several *Mesorhizobium* species and other soil-dwelling bacteria. Of these STEC-positive N-field taxa, only one species, *Zimmermannella faecalis*, first isolated from bovine feces, is not known as a soil-dwelling bacterium. STEC-positive soils were significantly depleted in taxa from the Xanthomonadaceae family relative to STEC-negative soils. It is worth noting that where significant taxa were identified between STEC-positive and STEC-negative soils, their RAs were generally well below 1%.

## DISCUSSION

While the soil benefits and microbial pathogen risks of using untreated dairy manure as a soil amendment have been previously observed, there is more to be learned about the longevity of these effects on the natural soil environment (7, 24, 32, 33, 36, 37). Here, we examined the effects of untreated dairy manure amendment on soil health and the microbiome, with particular focus on naturally occurring STEC, for more than one year. Metagenomic and soil health analyses were used to compare two fields from limited-mechanization farms in Ohio: field B, which was amended with untreated dairy manure, and field N, which was not amended with a BSAAO. The manure amendment was naturally contaminated with STEC, and STEC was identified in amended soils following amendment. While most microbiological impacts persisted through four weeks following amendment application and dissipated by six weeks post-amendment, soil physical and chemical impacts lasted for months, suggesting short-term risks and long-term benefits for using untreated dairy manure as a soil amendment on these farms. However, STEC detection was not limited to the weeks following amendment and was instead identified throughout the year in both fields, demonstrating that STEC contamination in produce fields is a complex and persistent problem regardless of the types of soil amendments used.

Soil amendments, such as manure, are added to soil to improve soil health, with a particular interest in increasing nitrogen availability for plants (1, 38). Indeed, field B had better soil health as indicated by several measurements, including organic nitrogen and estimated biological nitrogen. When taken together, soils from field B had significantly higher concentrations of more soil nutrients, namely, organic carbon and nitrogen, sodium, sulfur, boron, manganese, and zinc, throughout the year than field N, which had significantly higher phosphorus and calcium concentrations. In addition, field B had significantly higher water content than field N in the winter, particularly in the weeks following amendment application. This is consistent with previous research, as water holding capacity is one of the known benefits of BSAAO application (38). The increased soil moisture content persisted after tilling the amendment into the soil, suggesting benefits beyond as a soil cover, but disappeared during the summer months.

In this study, we consistently detected STEC in amended soils for the four weeks following amendment with untreated dairy manure. This effect was stronger in 2020, where all soil samples were STEC-positive, than it was in 2021. These differences in STEC detection could be due to sampling frequency in the days following amendment or to a higher concentration of STEC in the original manure in 2020. Notably, *E. coli* O-serogroup diversity was higher in the 2020 manure and soil samples, and STEC presence has been shown to be correlated with higher *E. coli* diversity both in this study and in our other work (data not shown). Previous studies have revealed that inoculation dose can influence STEC persistence in the environment, with higher doses corresponding to longer survival, and that concentration of STEC in manure varies based on cattle shedding rates and age of the manure (27, 37, 39). However, in this study, STEC was not quantified in the manure, and sampling was interrupted at about three weeks post-amendment in 2020, so it is unknown whether a potential difference in STEC bacterial load would have influenced how long STEC persisted in the soil.

Current language in the Food Safety Modernization Act Produce Safety Rule regarding the application of BSAAOs on agricultural soils recommends a waiting period of 90 days for crops not in contact with soils and 120 days for crops that come into contact with soils between amendment application and harvest, to prevent transfer of pathogens to produce (3). In this study, BSAAO-introduced STEC is no longer detected by six weeks after amendment—well within the recommended 90-day waiting period. Previous research has similarly noted a sharp decline in STEC populations by 30 days post-amendment (24, 37). Various timelines of STEC survival in soils have been reported, highlighting the complexities of environmental conditions that support or hinder pathogen survival (7, 8, 24, 37, 40). In some studies, STEC persistence far exceeded the 120-day recommendations, surviving months in cattle manure as well as soils and environments exposed to cattle manure (8, 24, 37, 40, 41). Laboratory-based studies demonstrate longer persistence of *E. coli* than field-based studies, but often fail to replicate the multiple environmental factors found in the field (23). Variation in *E. coli* survival times has been attributed to edaphic factors (such as soil moisture and pH), region, farming practices (amendment application rate and depth), weather (temperature and precipitation), and serotype grouping (23, 37, 42–44).

Previous research has focused on inoculated soil samples, and while this is important to understand STEC survival capabilities in various soil conditions, there are questions about how inoculated doses compare to natural conditions. We do not know how the levels of natural STEC contamination in this study compare with those used in inoculation studies, except that they are likely orders of magnitude lower (45, 46). This study of natural contamination does have limitations not found in a controlled experiment, including an unknown concentration of pathogen added to the soil, a non-uniform distribution of pathogen, and missing farm practice metadata (some information was confidential). However, it does offer useful insights into the natural environment that are not necessarily gained from experimental conditions. For example, the identification of multiple *stx* subtypes suggests that the pathogen was added to the soil as part of a consortium of STEC in the manure. Some previous research has shown differential persistence of pathogenic and non-pathogenic strains of *E. coli*, as well as of STEC strains belonging to differing O-serogroups when inoculated as a consortium in animal feces, but we are not aware of research looking at population fluctuations based on *stx* type (8, 13). Natural samples also contain other microbes, including non-STEC *E. coli*, that co-exist with the STEC in their native reservoir and may also impact STEC survival in the soil environment. In both years, amendment significantly increased the diversity of *E. coli* in the soil, and reflecting the amendment itself, the increase was higher in 2020 than in 2021. It is unknown whether the composition or diversity of the *E. coli* community influences the longevity of STEC in the soil.

STEC identification was not limited to soils amended with the STEC-containing BSAAO. Both the soil from the unamended field and soil from outside the four weeks post-amendment were found to harbor STEC at the same rate. This suggests that after the initial impacts of the amendment had subsided, one field was not more likely to be contaminated than the other. STEC contamination at these time points was not widespread compared to those samples taken immediately following amendment. In the four weeks post-amendment, STEC was consistently identified in the soil and found in at least two of the three replicate samples at any given time point. By contrast, when STEC was detected outside of the four weeks post-amendment, it was often only in a single replicate. The diversity of *stx* subtypes, particularly in field N, and limited distribution suggests that these additional STEC-positive samples are due to independent contamination events, rather than latent, low-level STEC in the soil; however, more data would be necessary to confirm this hypothesis. Since samples were taken several weeks apart at this point in the study, we were unable to determine how long the effects of each of these contamination events lasted.

We were interested in determining environmental and ecological factors that may correlate with STEC presence, particularly in those instances of STEC-positive samples outside of the four weeks post-amendment. STEC detection was significantly correlated with increased soil moisture, in accordance with previous studies that have identified soil moisture as positively impacting *E. coli* survival in soils (7, 25, 36, 37, 43). However, in this study, the moisture-STEC correlation was only statistically significant when amended soils were included and did not hold for STEC-positive samples in unamended soil or outside the four weeks post-amendment. The presence of STEC in the soil was also correlated with decreased concentrations of calcium, magnesium, organic nitrogen, and lower cation exchange capacity (CEC). These soil factors have been previously identified as influential to the soil microbiome, as well as on foodborne pathogen or *E. coli* persistence (47–49). Unlike the results presented here, the earlier studies reported positive correlations between calcium, magnesium, and CEC with persistence (47, 49–51).

A meta-analysis by Tran et al. on environmental factors that drive pathogen persistence in manure-amended soils identified temperature as the most significant factor in *E. coli* reduction under field conditions (23). In the current study, temperature was only correlated with STEC detection in the four weeks post-amendment, where it was likely a result of manure application taking place in winter. However, air and soil temperature were two of several environmental factors that influenced the shifts in the soil microbiome throughout the seasons. While the Tran et al. meta-analysis did not consider the role of the soil microbiome (23), other studies have identified native microbes as important factors in hindering pathogen survival (42, 43, 51–53). Here, we demonstrate that the soil microbiome shifts throughout the year in response to changes in seasonal environmental factors and even changes over a multi-year time span. The impacts of season on agricultural microbiomes and pathogen presence have been observed previously (37, 54, 55). Changes in the microbiota of the amended and unamended fields mirrored one another, and the two fields were rarely significantly different from one another, suggesting that the role of the environment on the microbiome was greater than that of the amendment practices, in line with previous studies (56). Information about other farming practices and their timing was limited, so it is unknown how such practices could have influenced the development of the soil microbiome or STEC presence. Indeed, outside of the initial application of STEC-contaminated manure amendment, neither field was more susceptible to the presence of STEC in the soil, as it was detected at the same rate in both.

The influence of the microbiome on STEC survival is described in the literature, with studies showing improved STEC survival in soils with reduced or eliminated microbiomes (42, 51). However, it is not known whether specific features of the microbiome are correlated with STEC presence or persistence. Using metagenomic data of the natural soil microbiomes, we sought to uncover how the soil microbiome correlates with the presence of STEC. The overall soil microbial community was not significantly correlated with STEC presence using either alpha or beta diversity measures. However, when specifically considering the *E. coli* community, a higher O-serogroup richness correlated with STEC presence. In amended soils, differentially abundant taxa reflected the manure microbiome, with species such as *Pseudomonas caeni*—the most abundant taxa in the manure amendment—emerging as the most significantly enriched taxa in amended soils vs. unamended soils. We were particularly interested in identifying taxa correlated with STEC in the unamended soils but were unable to find any that would suggest a source of STEC or could serve as a useful biomarker. In field B, no significantly differentially abundant taxa could be found in the STEC-positive soil samples relative to STEC-negative soils outside the four-week amendment timeframe. Considering the STEC-positive samples may have been the result of different sources of contamination, there may not be a consistent source that can be identified in this small number of samples. In field N, several taxa were identified as being significantly different in relative abundance between STEC-positive and STEC-negative soils. These taxa, many from the Xanthomonadaceae, Phyllobacteriaceae, and Methylobacteriaceae families, were all plant- or soil-related taxa that did not give indications about the potential source of STEC in these samples. The timing of spikes in the detection of several STEC-related genes aligned with summer and the start of harvest and may be due to increased activity in the fields around this time, although the details of potential routes or sources of STEC remain unknown.

This study offers a field-based perspective on the dynamics of naturally occurring STEC in manure-amended soils, providing valuable insight into the persistence of STEC and the long-term effects of BSAAOs on soil health. While STEC introduced through untreated dairy manure largely dissipated within the FDA-recommended 90- to 120-day preharvest interval, its sporadic presence throughout the year, even in unamended soils, highlights the complexity of contamination pathways in real-world agricultural systems. Critical questions remain about the ecological and microbial factors that govern STEC survival, particularly beyond the immediate post-amendment period. The lack of clear microbial biomarkers for STEC presence, along with the year-to-year variation in soil microbial communities, suggests that long-term, high-resolution studies are needed to fully understand how soil microbiomes evolve and influence food safety risk. As shifting agricultural practices alter soil environments, understanding these changing interactions will be essential for developing science-based management strategies that protect both crop productivity and public health.

## MATERIALS AND METHODS

### Field information and sample collection

Soil and manure samples were collected from limited-mechanization, small specialty crop farms in northeast Ohio. These farms grew a variety of commodities on a staggered schedule and had animals, including work animals, on the farm or on neighboring farms. Two farms were selected for their proximity to one another (approximately two miles apart) and their amendment practices. The farms were enrolled through voluntary agreement with the farmers and were blinded for the purposes of this study. One farm, field B, used bedding pack manure from dairy cattle as a soil amendment, while the other, field N, did not use a BSAAO and had not done so for the past 4 years. The bedding pack manure consisted of raw dairy heifer manure combined with corn stalks or straw. The manure pack accumulated in a pile over several months of use by live animals in the housing facility before being spread on the field in late winter. The manure amendment was applied to field B’s field in early February of 2020 and late February of 2021. Tilling of the soil took place in late March on field N and in mid-April on field B, followed by the first planting. Harvesting on both farms started in late June. Weather conditions were similar at the farms, and daily meteorological data were collected from the College of Food, Agricultural, and Environmental Sciences Weather System Wooster station (https://weather.cfaes.osu.edu/stationinfo.asp?id=1), the closest weather station to the sampled areas (within 24 km of the fields). Weather data included measurements of precipitation, air temperature, relative humidity, solar radiation, wind direction, wind speed, and soil temperature. Three replicate soil samples were collected at each time point, with each replicate sample being a composite of five subsamples from different locations in the field, resulting in approximately 2 kg of soil. Samples were shipped overnight, on ice, to the FDA facility in Laurel, MD, USA.

### Sample timeline

Sampling began in January 2020, with samples collected every two to three days following amendment, but was interrupted in March 2020 by the COVID pandemic three weeks after amendment. Sampling was restarted in January 2021, with samples collected from the amended field every 2 weeks, tapering off to monthly intervals (with more intensive sampling every three days following tilling of the fields) for one year after amendment was applied. Sampling from the unamended field occurred at approximately every other sampling event. Pre-amendment soil was collected from both fields in the month and the day before amendment application at field B. Soil amendment was collected from the field the day after application. Details for the timing of sample collection are listed in Table 1.

### Sample processing and enrichment

Soil and manure samples were manually homogenized and portioned for various analyses. Soil enrichment was completed using a modified FDA BAM method for *E. coli* enrichment (57). Briefly, 50 g of soil were incubated in 225 mL modified buffered peptone water with pyruvate at 37°C for five hours followed by the addition of vancomycin, acriflavine, and cefsulodin and incubation at 42°C for 19 hours. Following incubation, 2 mL of the resulting enriched culture was centrifuged, and the pellet stored at −80°C until DNA extraction. For culture-independent soil microbiome analysis, a 5 g portion of soil was stored at −80°C until DNA extraction.

### Soil physical/chemical analysis and moisture content

Gravimetric water content was determined by weighing approximately 10 g of soil before and after drying at 115°C for at least 24 hours. Soil and manure biophysical and chemical properties were measured by outside laboratories: the University of Maine Analytical and Maine Soil Testing Lab (Orono, ME, USA) for 2020 samples and Brookland Labs (New Bremen, OH, USA) for the 2021 samples, both using Mehlich III extraction methods. Full soil health analyses (measuring pH, organic carbon, organic nitrogen, IR gas analysis of soil respiration, nitrate, ammonium, P, K, Ca, Mg, Na, S, B, Fe, Mn, Cu, Zn, cation exchange, loss on ignition, and soluble salts) were obtained prior to amendment, one day after amendment, and the day after tilling. The pH (1:1 H_2_O), water-soluble carbon, and water-soluble nitrogen were measured eight additional times throughout the year (approximately every two months). A Student’s *t*-test was used to determine the statistical significance of mean soil measurements between sample groups.

### DNA extraction and sequencing

DNA extractions were performed using the Qiagen DNeasy PowerSoil Kit and the DNeasy PowerMax Kit (Qiagen, Germantown, MD, USA) for the enrichment pellets and unenriched soil samples, respectively. The protocol was followed as per manufacturer’s instructions, except for an increase in vortexing time from 10 to 15 minutes with beads. DNA concentration and purity were confirmed using a NanoDrop. When necessary, an additional cleanup was performed using the Zymo DNA Clean and Concentrator Kit (Zymo Research Corporation, Irvine, CA, USA).

Sequencing libraries were prepared using the Nextera DNA Flex Library Prep Kit (Illumina, San Diego, CA) and were sequenced with 150 bp paired-end sequencing on an Illumina NextSeq platform. Metagenomic sequencing was performed on a total of 160 samples, comprising 102 soil samples from field B, 48 soil samples from field N, and 10 manure samples. Both culture-independent (CI) and *E. coli* enrichments were sequenced for each sample. Enriched samples yielded an average of 22.7 million reads ranging from 10.1 million to 77.3 million, with one outlier at 198.7 million reads (Table S6). The CI samples had an average of 44.9 million reads (range 22.7 to 110.3 million reads) (Table S6).

### Sequence quality control, taxonomic identification, and *E. coli* characterization

Sequence reads were quality controlled using FastQC 0.11.8 (58). Sequences were trimmed for quality and removal of adapter sequences using the NexteraPE adapter file in Trimmomatic 0.38 (59). Microbial taxonomy was determined using custom C ++ programs and k-mer signature databases (github.com/mmammel8/kmer_id). Briefly, the 30 bp k-mer database is built from sequences of the RefSeq whole genomes plus WGS assemblies of species not included in the RefSeq. Counts for each taxon were tallied by exact matches in the reads and normalized by dividing the number of k-mers representing that taxon in the database. Relative abundances were determined by dividing the counts of each taxon by the total count. Confirmation of low-level species identifications was performed using BLAST analysis of the reads. The custom k-mer database used for bacterial identification contained 5,900 target entries, each with an average of 40,000 (range 44–80,000) unique k-mers.

Characterization of the *E. coli* community included molecular serotyping and virulence gene characterization. Briefly, the metagenomic reads were used in BLAST sequence homology searches against publicly available sequences in privately curated databases consisting of *E. coli* virulence factor genes and O-serogroup determinant genes (*wzm*, *wzt*, *wzx*, and *wzy*) (60, 61). Abundances of specific virulence factors and serogroups were inferred by tallying reads that best matched a specific virulence factor or serogroup allele with a minimum of 90% identity and 50 bp length. Ongoing research in our lab on *E. coli* diversity, through the analysis of publicly available WGS data, has identified numerous novel O-serogroups not included in the official *E. coli* O-serogroup panel (data not shown). These novel O-serogroups were included here to increase the sensitivity of *E. coli* detection and have been given provisional designations based on the determinant genes identified: OMT for *wzm* and *wzt* or OXY for *wzx* and *wzy. E. coli* diversity was determined by the number of identified O-serogroups. The *E. coli* core microbiome was determined by considering the occurrence of each O-serogroup at each time point as well as the consistency of its occurrence in the sample replicates. The proportion of occurrences at each time point was calculated by dividing the number of replicates in which the O-serogroup was found by the total number of replicates (three for soil samples and five for manure samples). For an O-serogroup to be considered part of the “core,” it had to occur in at least 66% of the replicates within a time point in at least 50% of the time points.

### Statistical analyses

Correlation between STEC presence and environmental data was inferred using a rank biserial correlation and a *t*-test to infer the significance of the correlation. The Wilcoxon rank sum test was used to compare median Shannon diversity, number of taxa, or number of serogroups between sample groups based on amendment use or STEC presence. Statistical analyses of correlations between O-serogroup richness and environmental measurements were performed using Spearman’s correlation. A Fisher’s exact test was used to determine the significance of the differential numbers of occurrences of individual O-serogroups or virulence genes between groups of samples. Statistical analyses were performed using R Statistical Software (version 4.0.4)(62).

### Microbial community analysis

Only species with a relative abundance of at least 0.02% were included in species richness and diversity analyses to reduce noise. Relative abundances of subspecies identified were added to corresponding species to reduce complexity. Shannon diversity was calculated using the vegan package (version 2.6-2) for R, and statistical comparisons were performed using the Wilcoxon rank sum test (62, 63). Richness was calculated as the number of taxa with a relative abundance greater than 0.02%.

Beta diversity was analyzed using the Bray–Curtis dissimilarity matrix for pairwise comparisons of each bacterial community using the pairwise distances function from the sklearn.metrics (version 1.3.2) module in Python 3.7.3 (64). The dissimilarity matrix was then input into the sklearn library (version 1.3.2) manifold function for non-metric multidimensional scaling (NMDS), and group clustering significance was determined by the skbio library (version 0.6.2) permutational multivariate analysis of variance (PERMANOVA) with 9,999 iterations (65, 66). Environmental factors were plotted on NMDS plots using the R envfit function in the vegan library (63).

ALDEx2 (R package ALDEx2 version 1.22.0) was used to infer significantly differentially abundant taxa between microbiome groups (67, 68). As with the other CI analyses, several taxa levels were included, down to the species level. Where subspecies were identified, reads were added together at the species-level designation. Taxa with an effect size of greater than 1 or less than −1 were considered significantly differentially abundant. Only taxa with an average of at least 0.02% relative abundance in at least 1 group were included.

## Data Availability

The sequencing data have been deposited in the NCBI database under the BioProject accession number PRJNA1193062 (Table S6). The program used for microbial community taxonomy can be found at https://github.com/mmammel8/kmer_id.

## SUPPLEMENTAL MATERIAL

Supplemental figures aem.00447-25-s0001.pdf Figures S1 to S4.

Supplemental tables aem.00447-25-s0002.xlsx Tables S1 to S9.

ASM does not own the copyrights to Supplemental Material that may be linked to, or accessed through, an article. The authors have granted ASM a non-exclusive, world-wide license to publish the Supplemental Material files. Please contact the corresponding author directly for reuse.
