# Supplementary material for: Long-term impacts of untreated dairy manure on the microbiome and Shiga toxin-producing Escherichia coli persistence in agricultural soil
Source: Appl Environ Microbiol. 2025 Aug 7;91(9):e00447-25. doi: 10.1128/aem.00447-25 (PMC12442374; doi:10.1128/aem.00447-25)
Supplement: Supplemental figures — Figures S1 to S4. [file aem.00447-25-s0001.pdf]

Figure S1: The number of O-serogroups for each replicate field B soil (blue), field N soil (green), and manure (orange) samples at each time point in A) 2020 and B)2021. STEC positive samples (solid circles) were correlated with higher O-serogroup diversity than STEC negative samples (open circles). Read counts for genes in each sample are shown in Table S4.

A

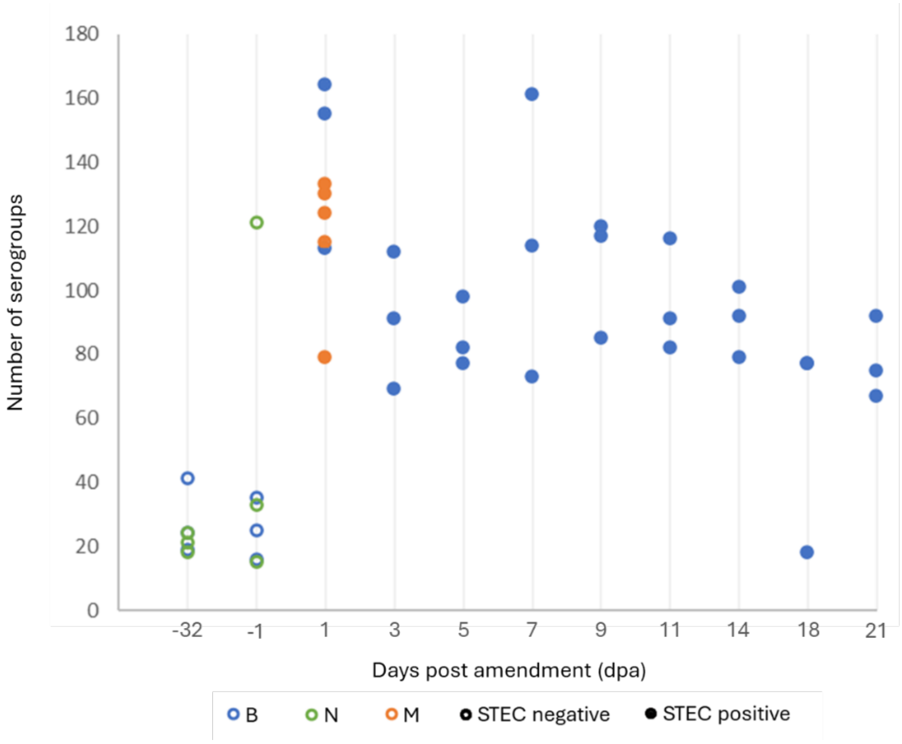

B

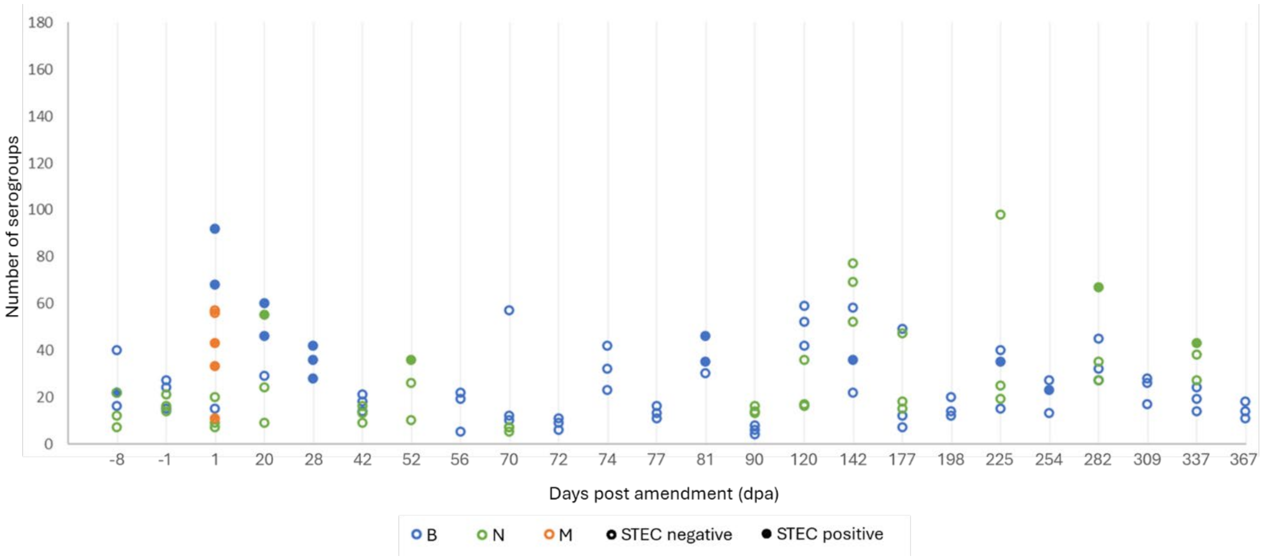

Figure S2: Summary of non-STEC, pathogenic *E. coli* genes in soil and manure samples showing the presence (+) and absence (-) of detected reads in (A) 2020 and (B) 2021. The genes encode virulence factors as follows: *pAA_aafA* aggregative adherence fimbriae subunit found on the pAA plasmid, *ipaH* invasion plasmid antigen H, *cdtB* cytolethal distending toxin subunit B, *pEAF_bfpA* bundle forming pilus subunit A found on the pEAF plasmid, *pEAF_perA* transcriptional regulator of the bundle forming pilus operon on the pEAF plasmid, LT-II subtype 2 of the heat labile toxin, *sta* heat stable toxin, *cnf* cytotoxic necrotizing factor, *hlyA* hemolysin, *papD* chaperone for P pilus biogenesis. Read counts for genes in each sample are shown in Table S7.

A

	pAA_aafA			*ipaH*			*cdtB*			*pEAF_bfpA*			*pEAF_perA*			LT-II			*sta*			*cnf*			*hlyA*			*papD*		
dpa	B	N	M	B	N	M	B	N	M	B	N	M	B	N	M	B	N	M	B	N	M	B	N	M	B	N	M	B	N	M
-32	-	-		-	-		-	+		-	-		-	-		+	+		-	-		-	-		-	+		-	-	
-1	-	-		-	-		+	+		-	+		-	+		+	+		-	-		-	+		-	+		+	+	
1	-		-	-		-	+		+	+		+	+		+	+		+	+		-	+		+	+		+	+		+
3	-			-			+			+			+			+			+			+			+			+		
5	-			-			+			+			+			+			-			-			+			+		
7	-			-			+			+			+			+			-			+			+			+		
9	-			-			+			+			+			+			-			+			+			+		
11	-			-			+			+			-			+			-			+			+			+		
14	-			-			+			+			+			+			-			+			+			+		
18	-			-			+			+			+			+			-			+			+			+		
21	+			-			+			-			+			+			-			+			+			+		

B

	pAA_aafA			*ipaH*			*cdtB*			*pEAF_bfpA*			*pEAF_perA*			LT-II			*sta*			*cnf*			*hlyA*			*papD*		
dpa	B	N	M	B	N	M	B	N	M	B	N	M	B	N	M	B	N	M	B	N	M	B	N	M	B	N	M	B	N	M
-8	-	-		-	-		-	-		-	-		-	-		+	-		+	-		-	-		+	+		+	-	
-1	-	-		-	-		+	-		+	-		+	-		-	+		-	-		-	-		-	-		-	-	
1	-	-	-	-	-	-	+	-	+	+	-	-	+	-	+	+	-	+	-	-	-	+	-	+	+	+	+	-	-	-
20	-	-		-	-		+	-		+	-		+	-		+	+		+	-		+	+		+	+		-	-	
28	-			+			+			-			-			+			+			+			-			+		
42	-	-		-	-		-	-		-	-		-	-		+	-		-	-		+	-		-	+		-	-	
52		-			+			-			-			-			-			-			+			-			+	
56	-			-			-			-			-			+			-			+			+			-		
70	-	-		-	-		+	-		-	-		-	-		+	-		-	-		+	-		+	-		-	-	
72	-			-			-			-			-			-			-			-			-			-		
74	-			-			-			-			-			+			-			-			-			-		
77	-			-			-			-			+			-			-			-			-			-		
81	-			-			+			-			-			+			-			-			+			+		
90	-	-		-	-		-	-		-	-		-	-		+	-		-	-		-	-		-	-		-	-	
120	+	-		-	-		+	+		-	+		-	+		+	-		-	+		+	+		+	+		+	-	
142	-	-		-	-		+	+		+	-		-	-		+	+		-	+		+	+		+	+		+	+	
177	-	-		-	-		-	+		+	-		+	+		+	+		-	+		-	+		-	+		-	-	
198	-			-			-			-			-			-			-			-			-			+		
225	-	-		-	-		+	+		-	-		-	-		+	+		+	-		+	+		+	-		-	-	
254	-			-			-			-			-			-			-			-			-			-		
282	-	-		-	-		-	+		-	-		-	-		-	+		-	+		-	-		-	+		-	+	
309	-			-			-			-			-			+			-			-			+			+		
337	-	-		-	-		-	-		-	-		-	-		+	-		-	-		-	-		+	-		-	+	
367	-			-			-			-			-			-			-			-			-			-		

Figure S3: Taxonomic distribution of the sample metagenomes showing taxa (as represented in the legend) that represent at least 5% relative abundance. Each replicate is shown within the sample time point and the timing of amendment and tilling is marked where applicable. (A) the microbiome of soil from the N field in 2020 (B) the microbiome of soil from the B field in 2020 (C) the microbiome of soil from the N field in 2021 (D) the microbiome of soil from B field in 2021 (E) the microbiome of manure in 2020 and 2021.

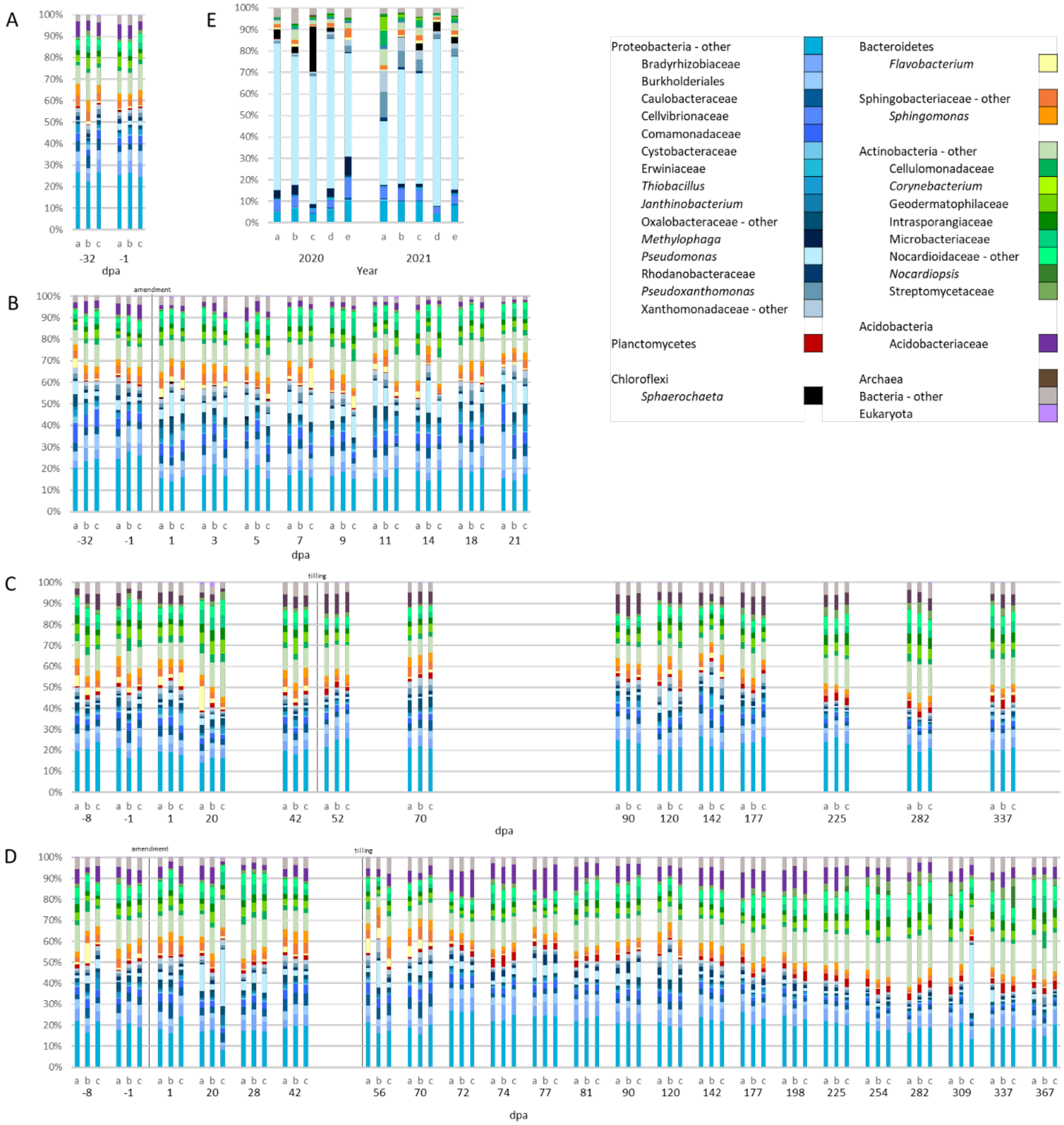

Figure S4: Summary of differential abundance analyses showing significantly enriched taxa in samples groups and their effect sizes. Relative abundances are shown as the average of all three replicates in a sample time point unless otherwise indicated. Taxa with an average relative abundance of less than 0.02 in both groups were excluded. (A) Differentially abundant taxa in

unamended vs amended soils in the B field in 2020. (B) Differentially abundant data in unamended vs amended soils in the B field in 2021. (C) Differentially abundant taxa in STEC positive vs negative samples in the N field in 2021.

A

Significant Taxa	Effect size	Avg Relative Abundance										
	dpa	-32	-1	1	3	5	7	9	11	14	18	21
	amendment	UA	UA	A	A	A	A	A	A	A	A	A
Pseudomonadaceae *Pseudomonas caeni*	3.17	0	0	1.6	0.9	2.68	1.29	2.39	0.46	1.87	0.38	0.32
Pseudomonadaceae *Pseudomonas litoralis*	2.55	0	0	0.1	0.11	0.15	0.05	0.07	0.03	0.08	0.03	0.02
Moraxellaceae *Acinetobacter lwoffii*	1.50	0	0	0.03	0.07	0.07	0.03	0.03	0.02	0.01	0.01	0
Pseudomonadaceae *Pseudomonas formosensis*	1.39	0.01	0	0.07	0.05	0.09	0.02	0.03	0.02	0.04	0.01	0.01
Sphingobacteriaceae *Pedobacter agri*	1.35	0.05	0	0.73	0.43	0.38	0.46	0.29	0.37	0.15	0.73	0.21
Pseudomonadaceae *Pseudomonas bauzanensis*	1.33	0	0.01	0.05	0.05	0.06	0.02	0.02	0.01	0.03	0.01	0.01
Microbacteriaceae	1.26	0.24	0.25	0.37	0.31	0.33	0.42	0.47	0.39	0.46	0.34	0.41
Pseudomonadaceae *Pseudomonas poae*	1.22	0	0	0.17	0.01	0.01	0.02	0.01	0.05	0.02	0.01	0.01
Acholeplasmataceae *Acholeplasma laidlawii*	1.21	0	0	0.18	0.04	0.14	0.05	0.08	0.01	0.02	0	0
Microbacteriaceae *Clavibacter michiganensis*	1.18	0.05	0.03	0.08	0.06	0.07	0.08	0.07	0.09	0.09	0.06	0.1
Porphyromonadaceae *Proteiniphilum acetatigenes*	1.18	0	0	0.04	0.03	0.06	0.02	0.02	0.01	0.01	0	0
Microbacteriaceae *Microbacterium chocolatum*	1.11	0.02	0.02	0.03	0.03	0.03	0.04	0.05	0.04	0.04	0.04	0.03
Microbacteriaceae *Microbacterium paraoxydans*	1.11	0.07	0.12	0.33	0.1	0.24	0.31	0.36	0.39	0.35	0.25	0.35
Microbacteriaceae *Microbacterium foliorum*	1.08	0.02	0.01	0.02	0.02	0.02	0.03	0.04	0.03	0.03	0.02	0.04
Microbacteriaceae *Microbacterium enclense*	1.07	0.02	0.02	0.04	0.04	0.03	0.05	0.04	0.05	0.05	0.03	0.04
Microbacteriaceae *Microbacterium*	1.06	0.34	0.37	0.48	0.46	0.49	0.64	0.86	0.59	0.78	0.55	0.55
Pseudomonadaceae *Pseudomonas congelans*	1.06	0.01	0	0.16	0.07	0.06	0.08	0.03	0.05	0.04	0.01	0.15
Microbacteriaceae *Microbacterium gubbeenense*	1.06	0.01	0.02	0.02	0.02	0.02	0.02	0.03	0.02	0.02	0.02	0.02
Caulobacteraceae *Brevundimonas subvibrioides*	1.05	0.08	0.05	0.12	0.13	0.1	0.1	0.1	0.12	0.11	0.13	0.18
Nocardioidaceae	1.03	1.27	1.05	1.39	1.59	1.68	1.62	1.77	1.48	1.57	1.57	1.48
Micrococcaceae *Arthrobacter gangotriensis*	1.00	0	0.01	0.06	0.04	0.14	0.06	0.09	0.08	0.11	0.06	0.18
Nocardioidaceae *Pimelobacter simplex*	1.00	0.14	0.13	0.16	0.17	0.18	0.18	0.2	0.16	0.17	0.17	0.16
Acidobacteriaceae	-1.44	3.61	5.05	1.67	2.58	2.79	1.98	1.83	1.89	1.69	1.87	0.92
Ectothiorhodospiraceae *Thioalkalivibrio*	-1.37	0.04	0.05	0.02	0.02	0.03	0.02	0.02	0.02	0.03	0.03	0.02
Acidobacteriaceae *Acidobacterium capsulatum*	-1.36	0.03	0.04	0.01	0.02	0.02	0.02	0.01	0.01	0.01	0.02	0.01
Kofleriaceae *Haliangium ochraceum*	-1.30	0.04	0.06	0.02	0.03	0.03	0.03	0.02	0.03	0.03	0.03	0.02
Sinobacteraceae *Nevskia ramosa*	-1.26	0.05	0.06	0.02	0.03	0.03	0.03	0.03	0.03	0.03	0.03	0.03
Anaeromyxobacteraceae *Anaeromyxobacter dehalogenans*	-1.24	0.06	0.07	0.03	0.04	0.05	0.04	0.03	0.03	0.04	0.04	0.03
Anaeromyxobacteraceae *Anaeromyxobacter*	-1.23	0.36	0.45	0.14	0.21	0	0.2	0.18	0.18	0.22	0.2	0.14
Ectothiorhodospiraceae *Spiribacter*	-1.19	0.02	0.03	0.01	0.02	0.01	0.01	0.01	0.01	0.01	0.01	0.01
Rhodocyclaceae *Aromatoleum aromaticum*	-1.13	0.07	0.09	0.04	0.05	0.06	0.05	0.04	0.05	0.05	0.06	0.04
Bdellovibrionaceae *Bdellovibrio*	-1.10	0.27	0.3	0.13	0.12	0.13	0.12	0.11	0.18	0.13	0.14	0.11
Chromatiaceae *Thiocapsa marina*	-1.09	0.03	0.05	0.02	0.03	0.03	0.03	0.02	0.02	0.02	0.02	0.01
Sinobacteraceae *Hydrocarboniphaga effusa*	-1.09	0.05	0.07	0.03	0.04	0.04	0.03	0.03	0.03	0.04	0.04	0.03
Sinobacteraceae *Singularimonas variicoloris*	-1.08	0.08	0.09	0.03	0.05	0.06	0.04	0.04	0.04	0.04	0.05	0.03
Rhodothermaceae *Rhodothermus marinus*	-1.08	0.03	0.03	0.01	0.02	0.02	0.01	0.01	0.01	0.01	0.02	0.01
Ectothiorhodospiraceae *Halorhodospira halophila*	-1.07	0.03	0.04	0.01	0.02	0.02	0.02	0.02	0.02	0.02	0.02	0.01
Burkholderiaceae *Cupriavidus necator*	-1.06	0.07	0.1	0.04	0.04	0.05	0.04	0.04	0.05	0.05	0.04	0.05
Chromatiaceae *Thioflavicoccus mobilis*	-1.05	0.03	0.04	0.02	0.02	0.03	0.02	0.02	0.02	0.03	0.03	0.02
Burkholderiaceae	-1.04	0.28	0.37	0.17	0.23	0.26	0.21	0.19	0.19	0.21	0.23	0.19
Ectothiorhodospiraceae *Alkalilimnicola ehrlichii*	-1.02	0.02	0.02	0.01	0.01	0.02	0.01	0.01	0.01	0.01	0.01	0.01
Gallionellaceae *Sideroxydans lithotrophicus*	-1.02	0.03	0.04	0.02	0.02	0.02	0.02	0.02	0.02	0.02	0.02	0.02
Rhodanobacteraceae *Rudaea cellulosilytica*	-1.01	0.1	0.13	0.05	0.07	0.09	0.06	0.06	0.06	0.07	0.08	0.06

B

Significant Taxa	Effect size	Avg Relative Abundance				
	dpa	-8	-1	1	20	28
	amendment	UA	UA	A	A	A
Pseudomonadaceae *Pseudomonas caeni*	2.52	0.00	0.00	0.04	0.15	0.39
Pseudomonadaceae *Pseudomonas litoralis*	2.30	0.00	0.00	0.02	0.02	0.05
Pseudomonadaceae *Pseudomonas syringae*	1.86	0.09	0.02	0.42	0.65	1.32
Pseudomonadaceae *Pseudomonas marginalis*	1.51	0.13	0.00	0.04	2.05	0.29
Microbacteriaceae *Microbacterium oxydans*	1.50	0.00	0.00	0.06	0.08	0.08
Micrococcaceae *Arthrobacter*	1.39	0.09	0.07	0.13	0.25	0.19
Microbacteriaceae *Leifsonia xyli*	1.35	0.02	0.01	0.02	0.02	0.03
Pseudomonadaceae *Pseudomonas orientalis*	1.34	0.01	0.00	0.05	0.02	0.02
Micrococcaceae *Paeniglutamicibacter gangotriensis*	1.31	0.00	0.01	0.01	0.06	0.20
Pseudomonadaceae *Pseudomonas trivialis*	1.23	0.01	0.00	0.01	0.04	0.02
Corynebacteriaceae *Corynebacterium maris*	1.22	0.01	0.01	0.03	0.02	0.06
Micrococcaceae *Arthrobacter nicotinovorans*	1.21	0.02	0.01	0.05	0.08	0.06
Microbacteriaceae *Microbacterium foliorum*	1.21	0.02	0.01	0.04	0.05	0.06
Microbacteriaceae *Microbacterium hydrocarbonoxydans*	1.20	0.03	0.02	0.06	0.06	0.08
Corynebacteriaceae *Corynebacterium*	1.18	0.02	0.02	0.02	0.02	0.05
Hyphomicrobiaceae *Pelagibacterium halotolerans*	1.14	0.02	0.02	0.03	0.03	0.03
Nocardiaceae *Rhodococcus qingshengii*	1.14	0.01	0.01	0.17	0.49	0.22
Micrococcaceae *Arthrobacter phenanthrenivorans*	1.12	0.02	0.02	0.03	0.05	0.04
Microbacteriaceae *Clavibacter michiganensis*	1.11	0.06	0.06	0.07	0.11	0.12
Sanguibacteraceae *Sanguibacter keddieii*	1.10	0.06	0.05	0.17	0.22	0.25
Rhizobiaceae *Agrobacterium fabrum*	1.02	0.02	0.02	0.03	0.03	0.03
Erythrobacteraceae *Erythrobacter litoralis*	-4.39	0.06	0.07	0.00	0.00	0.00
Intrasporangiaceae *Serinicoccus marinus*	-2.83	0.03	0.03	0.00	0.00	0.00
Bacillaceae *Bacillus megaterium*	-1.54	0.04	0.07	0.01	0.04	0.01
Chitinophagaceae *Niastella koreensis*	-1.39	0.04	0.03	0.01	0.02	0.02
Bacillaceae *Bacillus pseudomycoides*	-1.29	0.02	0.03	0.00	0.01	0.01
Chitinophagaceae *Niabella aurantiaca*	-1.27	0.07	0.08	0.04	0.05	0.03
Chitinophagaceae	-1.26	1.96	2.25	1.13	1.33	0.71
Rhodocyclaceae *Methyloversatilis universalis*	-1.04	0.04	0.04	0.03	0.03	0.03
Methylococcaceae *Methylococcus capsulatus*	-1.00	0.02	0.02	0.02	0.01	0.01

C

Significant Taxa	Effect Size	Avg Relative Abundance																	
	dpa	-8	-1	1	20 (a,b)	42	52 (b, c)	70	90	120	142	177	225	282 (b,c)	337 (b,c)	20c	52a	282a	337
	amendment	Neg	Neg	Neg	Neg	Neg	Neg	Neg	Neg	Neg	Neg	Neg	Neg	Neg	Neg	Pos	Pos	Pos	Pos
Phyllobacteriaceae *Mesorhizobium*	1.51	0.17	0.21	0.17	0.15	0.19	0.33	0.18	0.2	0.18	0.16	0.19	0.29	0.23	0.34	0.31	0.3	0.3	0.3
Methylobacteriaceae *Methylobacterium radiotolerans*	1.27	0.05	0.05	0.04	0.04	0.04	0.06	0.04	0.05	0.04	0.04	0.06	0.06	0.07	0.06	0.05	0.08	0.06	0.11
Promicromonosporaceae	1.16	0.64	0.64	0.51	0.88	0.57	0.79	0.4	0.36	0.61	0.4	0.66	0.59	0.92	0.88	0.9	0.86	1.13	0.97
Cellulomonadaceae *Cellulomonas flavigena*	1.12	0.06	0.06	0.05	0.07	0.05	0.05	0.04	0.05	0.05	0.04	0.05	0.05	0.06	0.06	0.08	0.06	0.08	0.07
Phyllobacteriaceae *Mesorhizobium opportunistum*	1.11	0.04	0.04	0.04	0.03	0.04	0.06	0.04	0.05	0.04	0.03	0.05	0.06	0.05	0.07	0.05	0.06	0.05	0.06
Microbacteriaceae *Zimmermannella faecalis*	1.10	0.01	0.01	0.01	0.02	0.02	0.01	0.02	0.01	0.01	0.02	0.01	0.01	0.02	0.02	0.02	0.02	0.03	0.02
Propionibacteriaceae *Microlunatus phosphovorus*	1.09	0.05	0.05	0.04	0.05	0.05	0.05	0.04	0.04	0.05	0.04	0.04	0.06	0.07	0.07	0.06	0.05	0.08	0.1
Intrasporangiaceae *Terracoccus*	1.06	0.14	0.15	0.11	0.23	0.15	0.13	0.13	0.11	0.17	0.12	0.11	0.23	0.26	0.21	0.27	0.15	0.23	0.31
Streptomycetaceae *Streptomyces bottropensis*	1.05	0.03	0.03	0.02	0.03	0.03	0.03	0.02	0.02	0.04	0.03	0.05	0.07	0.08	0.04	0.09	0.05	0.05	0.05
Phyllobacteriaceae *Mesorhizobium ciceri*	1.04	0.03	0.04	0.04	0.03	0.04	0.07	0.04	0.05	0.04	0.03	0.04	0.06	0.05	0.07	0.05	0.06	0.06	0.05
Methylobacteriaceae *Methylobacterium extorquens*	1.04	0.08	0.17	0.07	0.12	0.12	0.12	0.07	0.07	0.08	0.08	0.07	0.08	0.12	0.11	0.07	0.16	0.18	0.18
Pseudonocardiaceae *Saccharomonospora xinjiangensis*	1.03	0.02	0.02	0.01	0.02	0.02	0.02	0.01	0.02	0.02	0.01	0.02	0.02	0.02	0.02	0.02	0.03	0.02	0.02
Nocardiaceae *Nocardia brasiliensis*	1.02	0.03	0.03	0.02	0.03	0.03	0.04	0.02	0.02	0.03	0.02	0.03	0.05	0.05	0.04	0.04	0.05	0.04	0.04
Nocardiaceae *Nocardia cyriacigeorgica*	1.01	0.01	0.01	0.01	0.01	0.01	0.02	0.01	0.01	0.01	0.01	0.02	0.02	0.02	0.02	0.02	0.02	0.02	0.02
Xanthomonadaceae *Stenotrophomonas maltophilia*	-1.12	0.17	0.2	0.23	0.13	0.2	0.12	0.26	0.17	0.67	0.47	0.88	0.13	0.14	0.1	0.12	0.1	0.08	0.09
Xanthomonadaceae *Pseudoxanthomonas spadix*	-1.08	0.05	0.07	0.07	0.04	0.06	0.04	0.1	0.07	0.08	0.08	0.06	0.04	0.03	0.04	0.03	0.04	0.03	0.03
Xanthomonadaceae	-1.05	0.84	1.12	1.27	0.58	0.96	0.58	1.68	1.14	1.56	1.48	0.93	0.5	0.53	0.52	0.42	0.6	0.5	0.52
Xanthomonadaceae *Lysobacter antibioticus*	-1.03	0.11	0.19	0.18	0.04	0.12	0.05	0.27	0.15	0.2	0.23	0.2	0.07	0.09	0.06	0.04	0.09	0.05	0.04
Burkholderiaceae *Paraburkholderia caryophylli*	-1.01	0.02	0.02	0.02	0.01	0.02	0.03	0.02	0.03	0.02	0.02	0.02	0.03	0.01	0.02	0.01	0.02	0.01	0.01
Sphingobacteriaceae *Solitalea canadensis*	-1.00	0	0	0	0	0	0	0	0.01	0.01	0.01	0	0	0	0	0	0	0	0
